# Identification of signature genes and subtypes for heart failure diagnosis based on machine learning

**DOI:** 10.3389/fcvm.2025.1492192

**Published:** 2025-04-14

**Authors:** Yanlong Zhang, Yanming Fan, Fei Cheng, Dan Chen, Hualong Zhang

**Affiliations:** ^1^ Department of Cardiology, Xingtai People's Hospital, Xingtai, Hebei, China; ^2^ Department of Pediatrics Hematology and Oncology, Xingtai People's Hospital, Xingtai, Hebei, China

**Keywords:** heart failure, machine learning, immune characteristics, subtypes, pan-cancer

## Abstract

**Background:**

Heart failure (HF) is a multifaceted clinical condition, and our comprehension of its genetic pathogenesis continues to be significantly limited. Consequently, identifying specific genes for HF at the transcriptomic level may enhance early detection and allow for more targeted therapies for these individuals.

**Methods:**

HF datasets were acquired from the Gene Expression Omnibus (GEO) database (GSE57338), and through the application of bioinformatics and machine-learning algorithms. We identified four candidate genes (*FCN3*, *MNS1*, *SMOC2*, and *FREM1*) that may serve as potential diagnostics for HF. Furthermore, we validated the diagnostic value of these genes on additional GEO datasets (GSE21610 and GSE76701). In addition, we assessed the different subtypes of heart failure through unsupervised clustering, and investigations were conducted on the differences in the immunological microenvironment, improved functions, and pathways among these subtypes. Finally, a comprehensive analysis of the expression profile, prognostic value, and genetic and epigenetic alterations of four potential diagnostic candidate genes was performed based on The Cancer Genome Atlas pan-cancer database.

**Results:**

A total of 295 differential genes were identified in the HF dataset, and intersected with the blue module gene with the highest correlation to HF identified by weighted correlation network analysis (*r* = 0.72, *p* = 1.3 × 10^−43^), resulting in a total of 114 key HF genes. Furthermore, based on random forest, least absolute shrinkage and selection operator, and support vector machine algorithms, we finally identified four hub genes (*FCN3*, *FREM1*, *MNS1,* and *SMOC2*) that had good potential for diagnosis in HF (area under the curve > 0.7). Meanwhile, three subgroups for patients with HF were identified (C1, C2, and C3). Compared with the C1 and C2 groups, we eventually identified C3 as an immune subtype. Moreover, the pan-cancer study revealed that these four genes are closely associated with tumor development.

**Conclusions:**

Our research identified four unique genes (*FCN3*, *FREM1*, *MNS1*, and *SMOC2*), enhancing our comprehension of the causes of HF. This provides new diagnostic insights and potentially establishes a tailored approach for individualized HF treatment.

## Introduction

Heart failure (HF) represents a multifaceted clinical syndrome and the final phase of cardiovascular disease (1, 2). It poses a significant global public health challenge that is evolving rapidly, and the mortality rate within 4–5 years is approximately 50% (3), causing a heavy social and economic burden (3). Recently, the application of microarray technology along with integrated bioinformatics analysis has facilitated the identification of potential key genes linked to various diseases, which may subsequently serve as biological indicators for diagnosis and prognosis (4–6). Nevertheless, our comprehension of the genetic mechanisms underlying HF pathogenesis is still highly limited. Consequently, for early diagnosis and targeted treatment of these patients, specific HF genes should be identified at the transcriptome level.

Cancer is also widely recognized as a common co-morbidity in HF, with estimates suggesting that 5%–25% of total fatalities may be linked to cancer (7–9). While the two diseases might seem distinct entities, awareness is growing that cancer and heart failure share several common features. Notably, various potential tumor biomarkers, including CA125 and human epididymis protein 4 (HE4), have demonstrated a strong ability to forecast outcomes in HF as well (10–13). Following a myocardial infarction, cardiomyocytes exhibit a strong stress response, resulting in the activation of the nuclear factor NF-kB (14), a significant mechanism that promotes tumor growth. This activation leads to the induction of genes involved in cell proliferation, survival, angiogenesis, and metastasis (15, 16). These findings could indeed align with this idea, and tumor biomarkers might generally indicate the advancement of pathways that have traditionally been associated with specific cancers, but they may also play a significant role in the progression of heart failure. However, alterations in the transcriptome of immune cells may influence the prognosis of HF. It is thought that ongoing inflammation and immune irregularities play a role in the disease mechanisms throughout the range of HF (17). Recent studies have shown that immune cells, including macrophages and T cells, play a crucial part in the advancement and evolution of heart failure. Activation of inflammatory T lymphocytes and accumulation of inflammatory macrophages in the heart have been associated with adverse outcomes in individuals with HF (18).

Currently, HF biomarkers such as brain natriuretic peptide (BNP) or its precursor N-terminal proBNP (NT-proBNP) have been broadly validated for HF diagnosis. However, these markers may show variable sensitivity and specificity across different demographic groups, including elderly patients, women, and those with obesity or renal failure (19). Moreover, research emphasizes that using a combination of biomarkers may offer superior predictive value than relying on a single marker (20). Consequently, integrating multiple biomarkers into the clinical evaluation of heart failure is particularly important. Recent research has shown that machine-learning algorithms have successfully been employed to analyze vast datasets consisting of clinical, laboratory, and biomarker information. In one study, a novel model combining several machine-learning techniques achieved an accuracy of 87% in predicting heart failure, significantly improving the prediction capabilities compared to traditional models based solely on clinical data (21). Consequently, we retrieved microarray datasets associated with HF from the Gene Expression Omnibus (GEO) database. Our objectives are multi-fold: to foster the integration of diverse data sources, enhance the accuracy of prediction models, accomplish robust validation of biomarkers, and construct a theoretical framework for the diagnosis and treatment of HF. We intend to achieve these goals by synergistically applying a variety of standard analysis techniques in tandem with machine-learning methodologies.

## Materials and methods

### Datasets and source

A study procedure flowchart is shown in Supplementary Figure 1. Data on mRNA expression, alterations in copy number, DNA methylation 450 K data, mutation data, and clinical information of 33 cancers from The Cancer Genome Atlas (TCGA) database were used. We retrieved gene expression profile data of three different HF cohorts (GSE57338, GSE21610, and GSE76701) from the GEO database, which is affiliated with the National Center for Biotechnology Information (NCBI) (https://www.ncbi.nlm.nih.gov). The following criteria were used to screen datasets. (1) The keyword “Heart Failure” was used to identify the microarray data utilized in this study. (2) Each dataset must encompass a minimum of four samples each from patients with heart failure and control subjects. (3) We only considered datasets that contained readily accessible expression information. Table 1 presents the characteristics of the datasets.

**Table 1:** Datasets used in this study.

GEO dataset	Platform	Disease	Sample		Year	References
			Control	Case		
GSE57338	GPL11532	Heart failure	136	177	6 May 2014	(22)
GSE21610	GPL570	Heart failure	8	30	30 April 2010	(23)
GSE76701	GPL570	Heart failure	4	4	11 January 2016	(24)

### Data processing and differential gene analysis

We performed log_2_ transformation and then normalized the raw count expression data using the function “normalizeBetweenArrays” in the R package “limma.” To ensure accuracy and consistency, we removed probes corresponding to multiple molecules and retained only the probe with the highest signal value for each molecule. Next, we merged the gene expression data from GSE21610 and GSE76701 into a new matrix as the training group. Following the merging of the two datasets, the function “ComBat” in the R package “sva” was then utilized to remove the batch effect. Robust multi-array averaging (RMA) was employed for background correction and imputation of missing values. The final merged dataset consisted of 313 samples including 177 patients with HF and 136 controls. To gain insights into the differences between heart failure and normal samples, we used the limma package and identified genes with adjusted *p*-values <0.05 and |log_2_(FC)| ≥ 0.5 as the differentially expressed genes (DEGs).

### Weighted correlation network analysis

The network created using the weighted correlation network analysis (WGCNA), in the R package “WGCNA,” was used to investigate gene modules that display relationships. The genes exhibiting the highest variance, specifically the top quarter from the GSE57338 dataset, were chosen as the input data to enhance the precision of the findings. The ideal soft threshold power was identified through the scale-free topology criterion and a topological overlap matrix was generated. Modules containing more than 200 genes were selected using the hierarchical clustering tree methodology. Finally, a correlation analysis of module traits was conducted to identify the most important one related to HF and complete the visualization of the signature gene network.

### Functional enrichment analysis

The R package “clusterProfiler” was utilized to perform enrichment analysis for biological functions in Gene Ontology (GO) and pathways in the Kyoto Encyclopedia of Genes and Genomes (KEGG). The GO categories encompass biological processes (BPs), molecular functions (MFs), and cellular components (CCs).

### Machine-learning models for feature selection and visualization

Three machine-learning algorithms were used to further identify candidate genes for the diagnosis of heart failure. First, to enhance prediction accuracy and interpretability, we employed least absolute shrinkage and selection operator (LASSO) regression to select key features from the training dataset (GSE57338). LASSO is a regression method designed for high-dimensional data. It introduces a penalty term to the least squares method, compressing some regression coefficients to zero, which achieves variable selection and improves the model's generalization capability (25). In this study, we used the “glmnet” package in R to perform LASSO regression, and optimal lambda parameters were determined using 10-fold cross-validation, with the Lambda.1se value corresponding to the minimum cross-validation error selected as the model's optimal value. Next, we performed support vector machine recursive feature elimination (SVM-RFE) analysis using the “e1071” packages, which was used to iteratively remove less significant features and determine the optimal variables (26) for the classification of cancer using only two types of data in feature extraction. Finally, the random forest (RF) algorithm was executed using the “randomForest” package for classification, regression, and feature selection by building multiple decision trees, aggregating their results, and providing a robust evaluation of feature importance while handling noisy data (27). The intersecting genes from LASSO, SVM-RFE, and RF were considered candidate hub genes for HF diagnosis.

### Validation of diagnostic model

Receiver operating characteristic (ROC) curves were constructed to determine the diagnostic value of the candidate genes. The area under the curve (AUC) was calculated to quantify their value, with an AUC value greater than 0.7 being considered the ideal diagnostic threshold. To validate the robustness of these core genes, we conducted an analysis of individual and combined genes using the merged dataset (GSE21610 and GSE76701) for external validation. We assessed the discriminatory ability of the diagnostic model using ROC curves once again.

### Recognition of distinct subtypes by unsupervised clustering

The “partitioning around medoid” approach was employed to recognize subtypes of patients with HF, utilizing the ConsensusClusterPlus package. When determining the ideal number of subtypes, we evaluated the cumulative distribution function (CDF) curve performance, consensus matrix, and changes in the area under the CDF curve, and ensured a steady cluster score exceeding 0.9.

### Gene set enrichment analysis (GSVA)

Functional improvements in various subtypes were assessed using the “GSVA” package. The gene sets (h.all.v7.5.symbols.gmt and c2.cp.kegg.v7.2.symbols) were obtained from the Molecular Signatures Database (MSigDB) database. We visualized important paths between groups using the R package “ComplexHeatmap.”

### Tumor microenvironment characteristics

To estimate the cell abundance of the tumor microenvironment (TME), we utilized a compendium of microenvironment genes related to specific immune cell subsets. GSVA was used to evaluate the enrichment of 24 types of tumor immune microenvironment cells. The immune checkpoints’ distribution was analyzed, and the ESTIMATE R package was utilized to determine the immune and stromal scores of the tumor tissue. We also analyzed the expression of immunomodulatory factors, including chemokines, immune inhibitors, and immune stimulators, and the major histocompatibility complex (MHC) in different subtypes.

### Somatic copy number alteration and single nucleotide variation analysis

The amplification and deletion of heterozygosity and homozygosity were considered to enhance the somatic copy number alteration (SCNA) of every gene, with more than 5% classified as high-frequency SCNA. To evaluate the connection between expression levels and SCNA, Pearson’s correlation was computed using the expression values alongside the copy number segment values for each gene. For the calculation of the single nucleotide variation (SNV) percentage, the mutation frequency of SNVs in the coding region of each gene was determined by the following formula: number of mutated samples divided by the number of cancer samples. An oncoplot illustrating the SNV was created using maftools.

### Prognostic pan-cancer analysis of the hub genes for HF

Cox regression analyses, both univariate and multivariate, were conducted to evaluate the associations of different variables with overall survival (OS) in the “survival” package. The generation of Kaplan–Meier curves, accompanied by the log-rank test, was carried out with the aid of the “survminer” package.

### Statistical analysis

Data analysis was conducted using R software (version 4.0.0). Differences between two groups were assessed using the Wilcoxon test. Correlations were assessed using Pearson's correlation test. We performed the “K-means” method to identify subtypes, based on the “ConsensusClusterPlus” package. The CDF curve, consensus matrix, relative alterations in the area under the CDF curve, and a consistent cluster score (>0.9) were considered when selecting the optimal subtype numbers. The diagnostic ability of each key gene was evaluated by the AUC score. The false discovery rate (FDR) was calculated using the Benjamin–Hochberg method to adjust the *p*-value. All statistical tests were two-sided and *p* < 0.05 indicated statistical significance.

## Results

### Identification of differentially expressed genes

**Figure 1:**
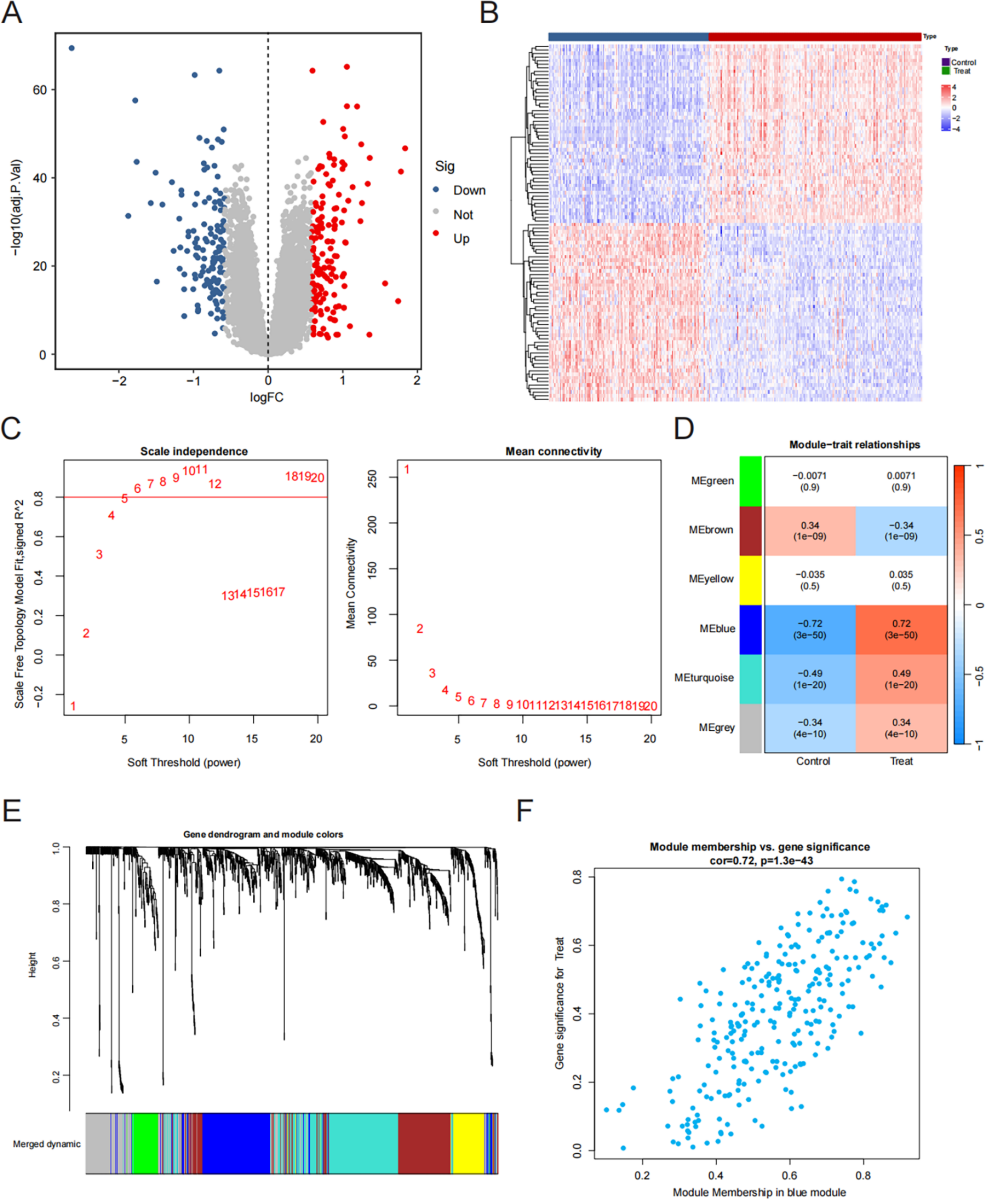
Identification of hub genes. **(A)** Volcano plot. Red dots are upregulated genes and blue dots are downregulated genes. **(B)** Heatmap plot. We showed the top 30 upregulated and the top 30 downregulated genes in the HF and control groups. **(C)** The scale-free fit index for soft-thresholding powers and mean connectivity. **(D)** Gene co-expression modules. **(E)** Gene and trait clustering dendrograms. **(F)** The correlation of the blue module and the HF group.

A comprehensive analysis utilizing the Lima package revealed 295 differential genes within the combined dataset, comprising 157 genes that were upregulated and 138 that were downregulated (Figures 1A,B).

### Weighted gene co-expression network analysis and key module identification

We utilized WGCNA to determine the most significant modules associated with heart failure. To approximate a scale-free topology for the network, a soft threshold power of 6 was applied (Figure 1C). Ultimately, we obtained six gene co-expression modules. The results showed that the blue module was significantly associated with heart failure and showed the strongest correlation (correlation coefficient = 0.72, *p* = 1.3 × 10^−43^) (Figures 1D–F). Consequently, we chose the blue module as the primary module for further analysis.

### Functional enrichment analysis of heart failure

**Figure 2:**
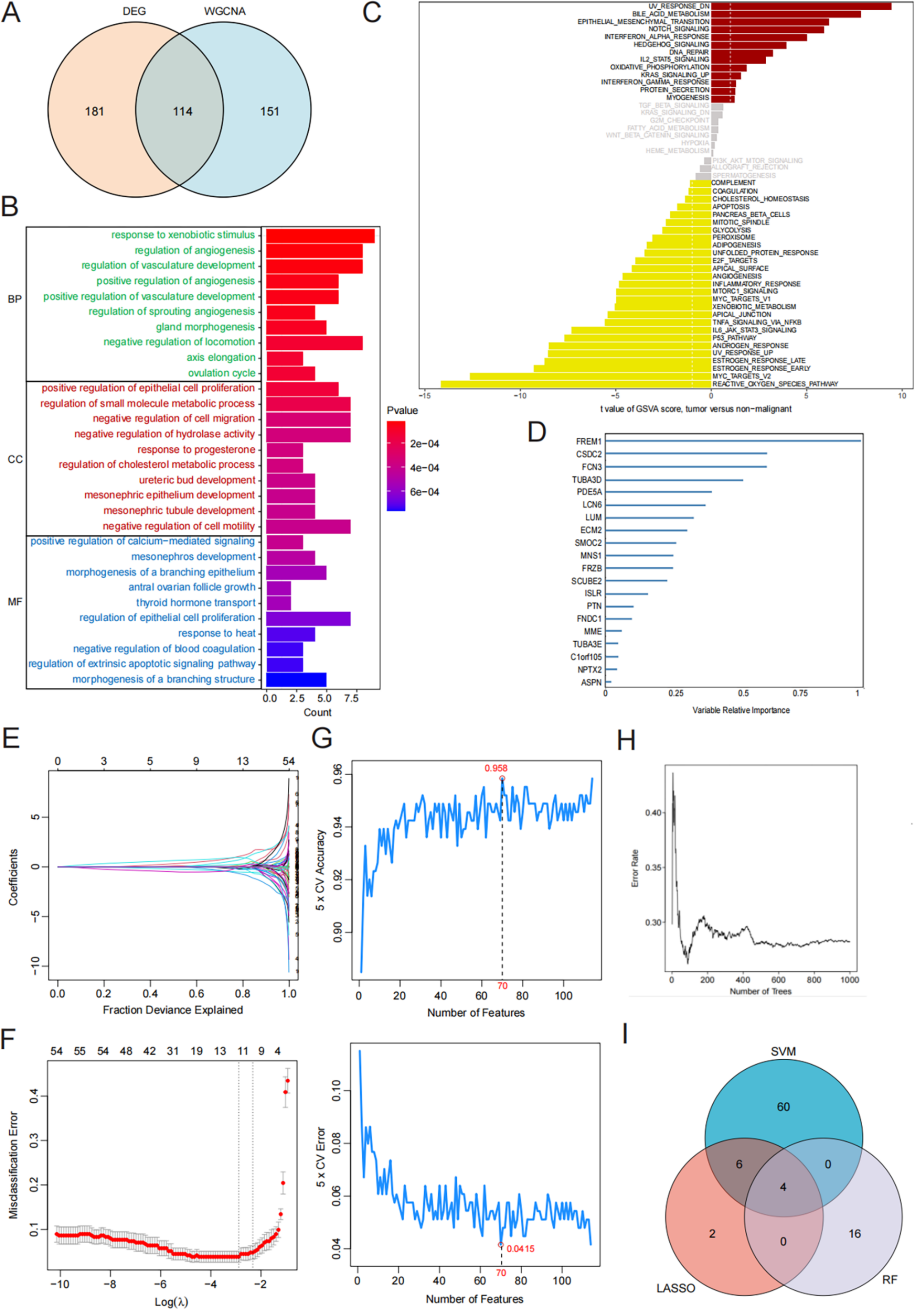
Machine learning for screening candidate diagnostic biomarkers for HF. **(A)** The intersection of DEGs and the blue module. **(B)** KEGG enrichment analysis results. **(C)** GO enrichment analysis results. **(D)** Biomarker screening in the RF model. **(E,F)** Biomarker screening in the LASSO model. **(G)** Biomarker screening in the SVM model. **(H)** Error rate for the data as a function of the classification tree. **(I)** Venn diagram showing the four candidate diagnostic genes identified via the previous three algorithms.

Afterward, we intersected DEGs with the blue module genes to obtain 114 key HF genes (Figure 2A). We performed GO enrichment analysis and the results showed that the key genes of HF mainly play a role in angiogenesis regulation and proliferation metabolism (Figure 2B). Further pathway enrichment analysis also found that they are mainly involved in the reactive oxygen species pathway, bile acid metabolism, and epithelial-mesenchymal transition (Figure 2C).

### Identification of hub genes via machine learning

Three machine-learning algorithms (RF, LASSO, and SVM-RFE) were employed to identify hub genes related to heart failure. The RF algorithm, based on the “randomforest” R software package, sorted genes according to their importance calculations (nrep = 1,000, which indicates that the number of iterations in the Monte Carlo simulation was 1,000; and nstep = 5, which indicates that the number of steps forward was 5). Figure 2H shows the relationship between the error rate and the number of classification trees. Finally, we selected the top 20 genes as potential candidates for heart failure (Figure 2D). We used the LASSO algorithm to further reduce the dimensionality and narrow down the range of feature genes. We used regularization methods in the model training to limit the complexity of the model and prevent overfitting. Finally, we retained 12 genes from 54 feature genes under the optimal log(*λ*) of −2.756 (Figures 2E,F). Through the 10-fold cross-validation, the minimum binomial deviation with optimal log(*λ*) was observed, indicating the 12 genes were optimally selected by LASSO. The SVM-RFE algorithm demonstrated the greatest precision, recognizing 114 genes while maintaining a steady precision score of 0.958 thereafter. To identify the optimal quantity of the hub genes, we selected the top 70 genes based on the SVM-RFE outcomes as our candidate genes (Figure 2G). Through the intersection of the outcomes from all three algorithms, we pinpointed four hub genes relevant to HF: *FCN3*, *FREM1*, *MNS1*, and *SMOC2* (Figure 2I).

### Diagnostic value assessment

**Figure 3:**
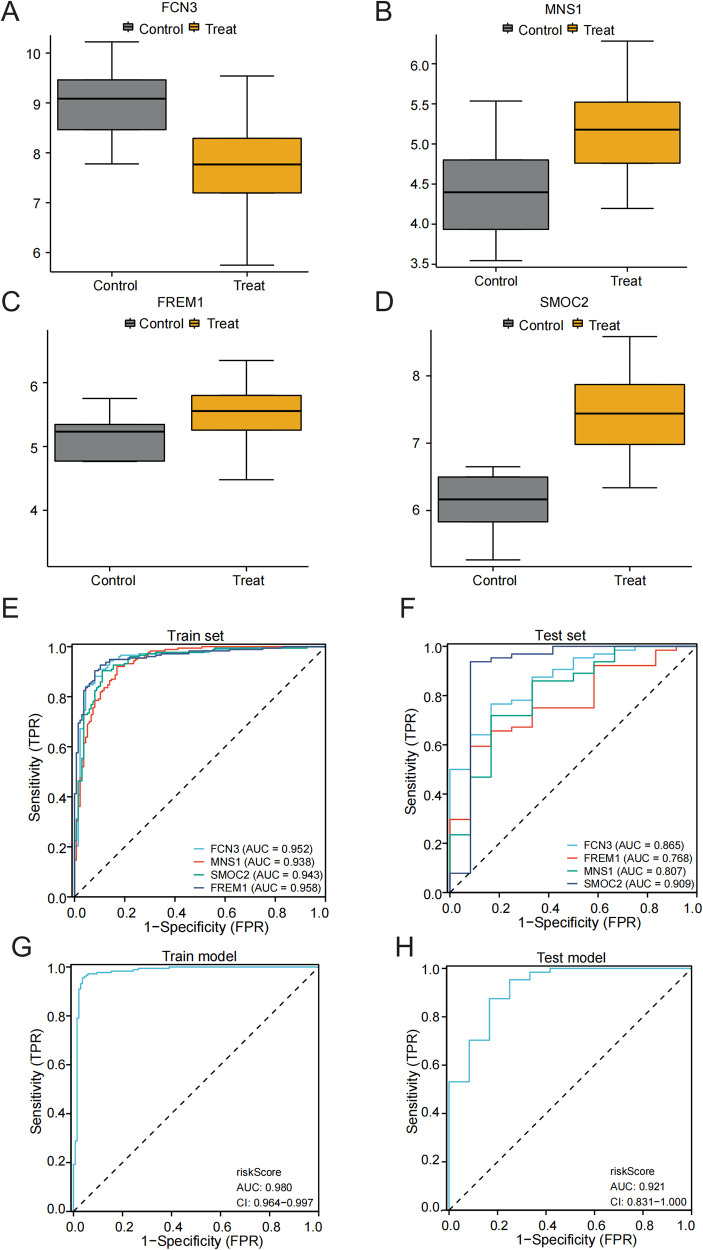
Validation of the diagnostic efficacy of characteristic genes. **(A–D)** Expression of hub genes in patients with HF compared to normal controls in GSE57338. **(E,F)** The ROC curve of each candidate gene in the training and test sets. **(G,H)** The ROC curve of the diagnostic model in the training and test sets.

We analyzed the expression of the four key genes, *FCN3*, *MNS1*, *SMOC2*, and *FREM1*, in the normal and HF groups (Figures 3A–D). Furthermore, we assessed the diagnostic significance of these genes utilizing ROC curves. In the training dataset (GSE57338), the areas under ROC curves for *FCN3*, *MNS1*, *SMOC2*, and *FREM1* were 0.952, 0.938, 0.943, and 0.958, respectively (Figure 3E). Furthermore, after merging and normalizing the data of GSE21610 and GSE76701 to be used as the validation set, the areas under ROC curves for *FCN3*, *MNS1*, *SMOC2*, and *FREM1* were 0.865, 0.807, 0.909, and 0.768, respectively (Figure 3F). All four gene signatures demonstrated high accuracy, with AUC values exceeding 0.7, indicating their predictive effectiveness. Nevertheless, when the four genes were integrated into a single diagnostic model, the AUC values for both the training and test datasets were 0.980 and 0.921, respectively (Figures 3G,H). This outcome demonstrated that the combination of these genes holds significant potential for diagnosis and could also function as valuable targets for both the prevention and management of HF.

### Identification of subtypes of patients with HF

**Figure 4:**
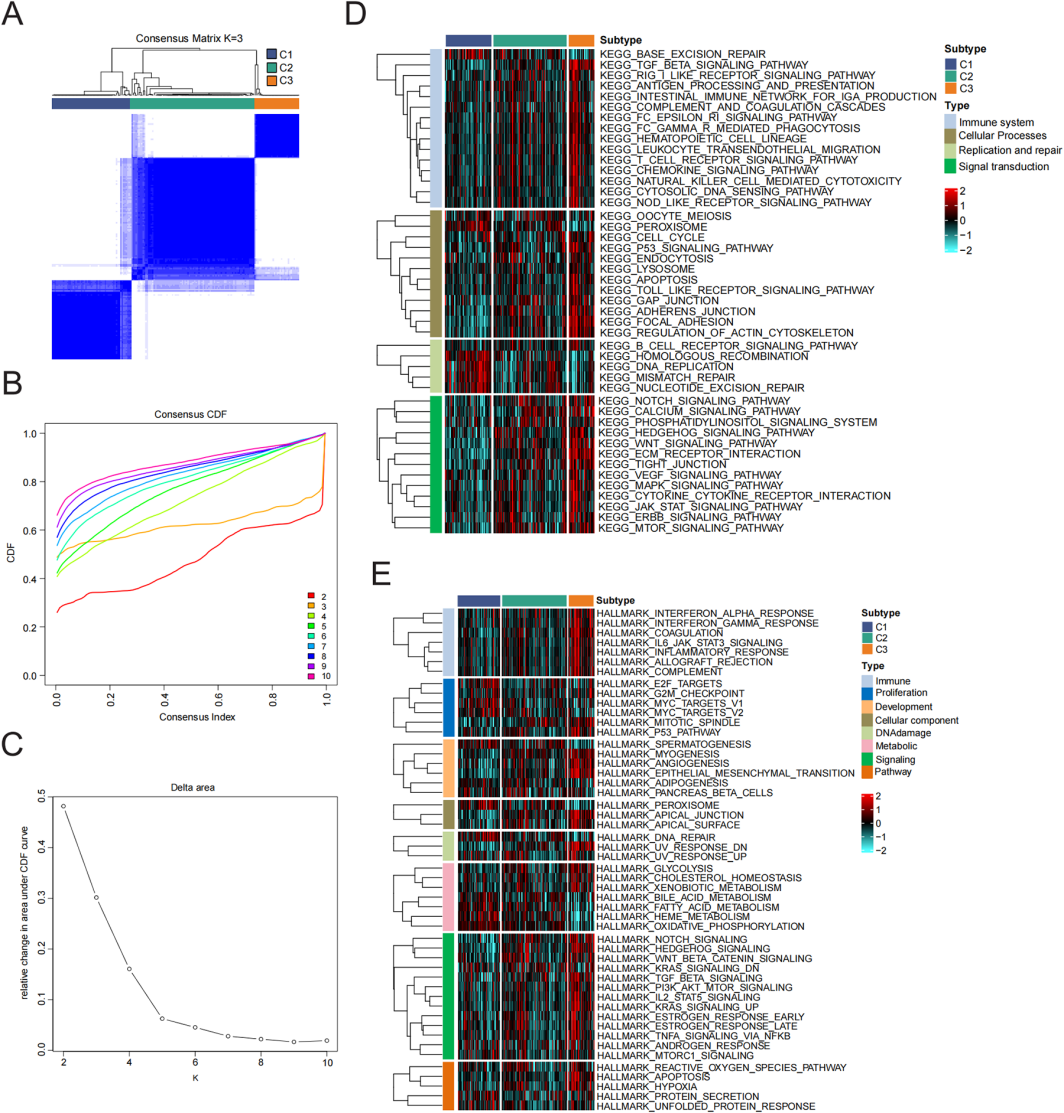
Identification and enrichment analysis of HF subtypes. **(A)** Consensus clustering matrix when k = 3. **(B)** Consensus CDF curves when k = 2–10. **(C)** Relative alterations in CDF delta area curves. **(D,E)** Signal pathway enrichment analysis of 50 hallmark gene sets and KEGG analysis.

To clarify the expression patterns associated with the immune microenvironment in HF, we utilized the consensus clustering algorithm to categorize the 176 HF tissue samples according to the four key genes. The similarity matrix was interpreted as the consensus matrix to determine the ultimate subtypes. Utilizing the results from consensus clustering, the CDF plot, the relative change in the area of the CDF curve, and the consistent cluster score, we determined that *k* = 3 was the best choice for categorizing 176 patients into three distinct subtypes (Figures 4A–C). Subsequently, we analyzed the biological functions and signaling pathways that were enriched, utilizing gene sets from the MSigDB, and conducted GSVA to assess the score for each patient. Compared to subtype 1 and subtype 2, patients with subtype 3 demonstrated the most immune activation (complement, interferon alpha response, and IL6-JAK-STAT3 signaling) and signaling-related processes (IL2-STAT5 signaling, TNFα signaling via NF-κB, and PI3K-AKT-mTOR signaling). Further KEGG enrichment analysis also confirmed these results (Figures 4D,E).

### Differentiation of immune characteristics between subtypes

**Figure 5:**
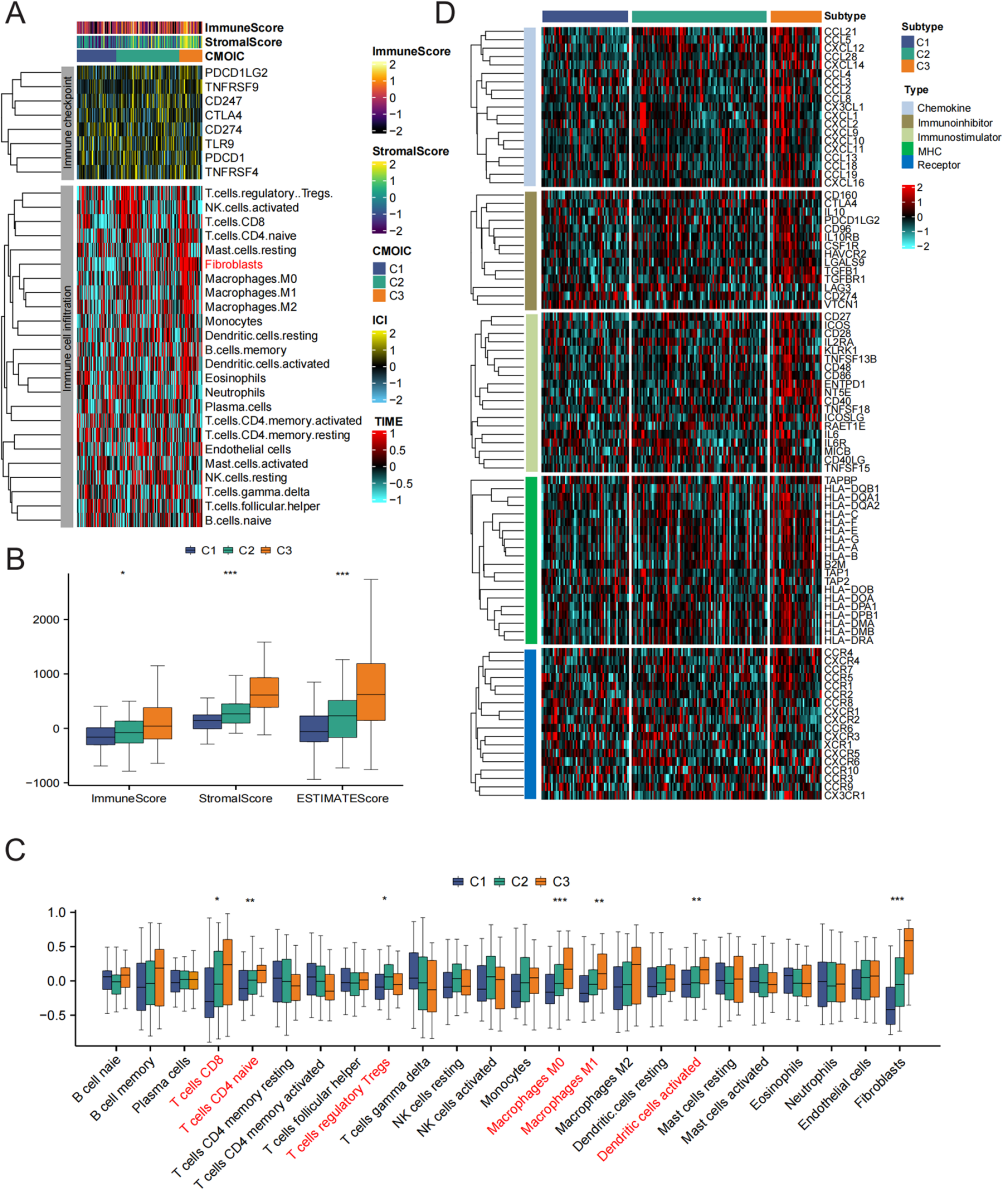
The immune characteristics of distinct HF subtypes. **(A)** Immune checkpoint expression and immune cell infiltration enrichment in the three subtypes. **(B)** The differences in ESTIMATEScore among the three subtypes. **(C)** The differences in infiltrated immune cells among the three groups. **(D)** Heatmap showing the differences in immune regulatory genes between subtypes.

Given the significant differences in immune processes among the clusters, we assessed the levels of infiltration by immune cells in the microenvironment and immune checkpoint expression in different groups. As shown in Figure 5A, the abundance of immune cell types was calculated using tumor immune estimation resource (TIMER) algorithms. The greatest infiltration of fibroblasts was noted in patients with subtype 3 compared to those in the subtype 1 and subtype 2 groups. In addition, a qualitative assessment of immune characteristics was conducted by comparing the immune scores, stromal scores, and estimate scores across each subtype. Patients with subtype 3 exhibited higher scores compared to the other subtypes (Figure 5B). Furthermore, CD8T cells, naive CD4T cells, regulatory T cells, macrophages, and activated dendritic cells (DCs) demonstrated elevated enrichment scores within the immune microenvironment of subtype 3 (Figure 5C). To investigate the variations in immune features across the different subtypes, we additionally assessed the expression levels of genes that regulate immunity within each subtype (Figure 5D). In immune microenvironment subtype3, all chemokines, immune inhibitor genes, immune stimulator genes, and MHCs were consistently highly expressed. Taking the results mentioned above into account, we ultimately classified subtype 3 as an immune subtype.

### Pan-cancer analysis of characteristic genes

**Figure 6:**
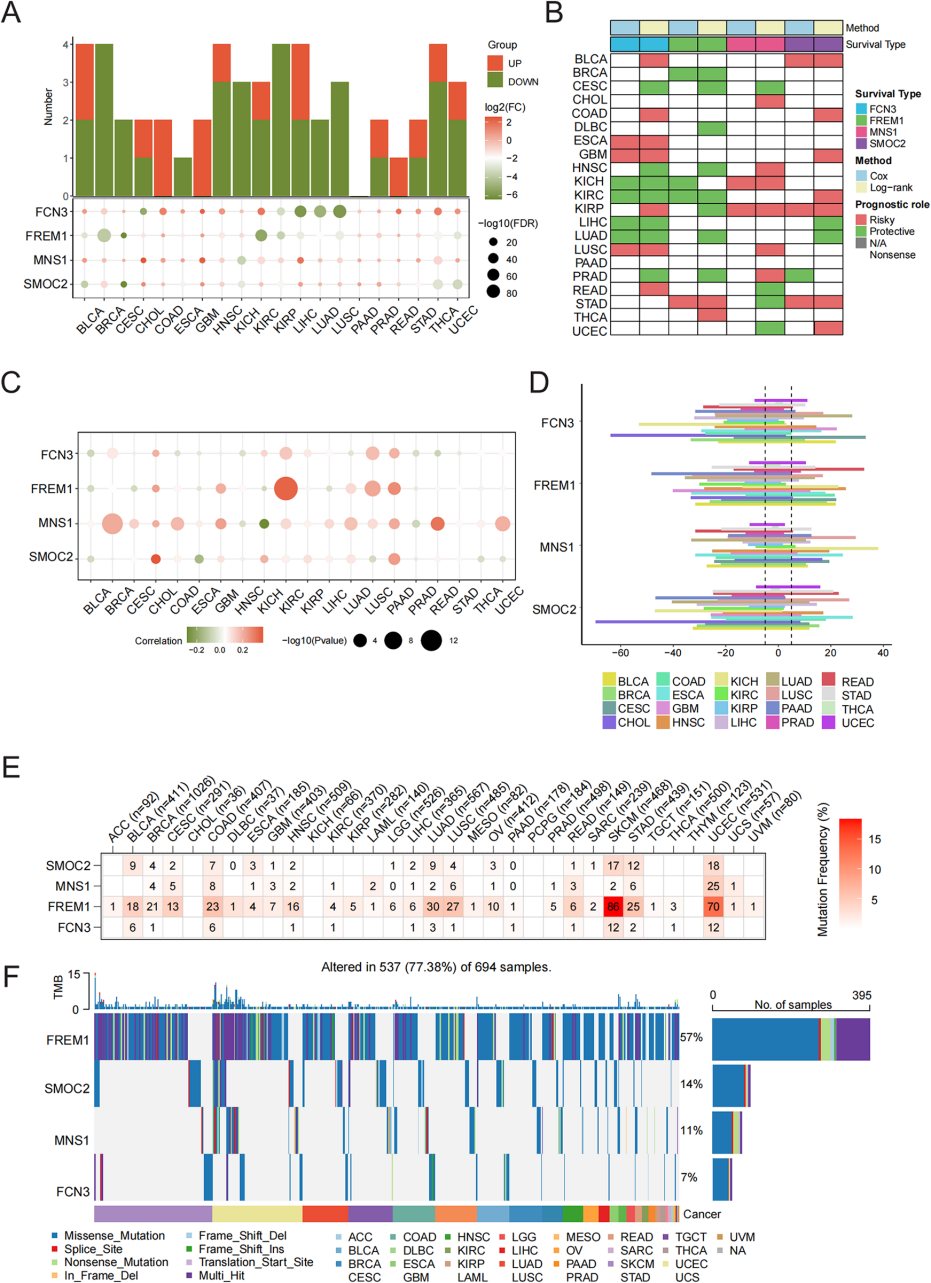
Pan-cancer validation of characteristic genes. **(A)** Histogram (upper panel) showing the number of upregulated or downregulated differentially expressed genes (DEGs) and the heatmap showing the fold change and FDR of four characteristic genes in each cancer. **(B)** Summary of the correlation between the four characteristic genes and survival of cancer patients. Red points indicate a high-risk prognostic factor, while green points indicate a protective factor. **(C)** Spearman's correlation analysis between copy number alteration and the expression of the four characteristic genes. **(D)** Somatic copy number variant analysis. **(E)** Mutation analysis of the four characteristic genes. Numbers represent the number of samples that have the corresponding mutated gene for a given cancer. **(F)** An oncoplot showing the mutation distribution of the four characteristic genes and a classification of SNV types.

Subsequently, we explored the varying expression levels of these four hallmark genes across 20 different cancer types and their corresponding normal tissues. It is important to highlight that the expression levels of *FCN3*, *FREM1*, *MNS1*, and *SMOC2* were markedly elevated in various types of cancerous tissues (Figure 6A). Subsequently, our goal was to concentrate on the signature genes that showed significant associations with patient survival across 33 distinct types of cancer. Our analysis revealed that all identified signature genes exhibited a strong correlation with the OS of patients in a minimum of three of these cancer categories (Figure 6B). For copy number analysis, the results showed that general somatic copy number alterations occurred at high frequencies (>5%) in most cancer types (Figure 6D). *FCN3*, *FREM1*, *MNS1,* and *SMOC2* presented diverse somatic copy number alteration profiles. We further investigated how SCNA influences gene expression and found the expression of *FCN3*, *FREM1*, *MNS1,* and *SMOC2* was significantly positively associated with SCNA in most cancers (Figure 6C), which indicated that aberrant SCNA of a gene might contribute to the progression of various cancers. In the mutation analysis, the main variant type was missense mutation. The mutation percentages for *FCN3*, *FREM1*, *MNS1,* and *SMOC2* were 7%, 57%, 11%, and 14%, respectively (Figure 6F). As shown in Figure 1D, *FREM1* had higher SNV frequencies in skin cutaneous melanoma (SKCM) and uterine corpus endometrial carcinoma (UCEC) (Figure 6F).

## Discussion

In this research, datasets from HF microarrays were acquired from the GEO database, leading to the identification of DEGs. Subsequently, we chose significantly modular genes using WGCNA. Three distinct machine-learning models using the LASSO, RF, and SVM-RFE techniques, respectively, were employed to identify hub genes associated with HF. Ultimately, a total of four genes (*FCN3*, *FREM1*, *MNS1*, and *SMOC2*) exhibited significant co-expression in individuals suffering from heart failure. These discoveries could enhance our comprehension of the mechanisms that drive the development of HF.

Current research on biomarkers for HF plays a critical role in understanding the pathophysiology and guiding clinical decisions. In particular, natriuretic peptides are well-established in clinical practice for diagnosing and prognosticating heart failure. However, they do have limitations related to specificity and can be influenced by various factors such as renal function, age, and obesity (20, 28). Studies have shifted focus toward novel biomarkers, such as soluble suppression of tumorigenicity 2 (sST2), high-sensitivity troponin, and growth differentiation factor 15 (GDF-15), which show promising prognostic capabilities in both acute and chronic heart failure settings (29, 30). In our research, we specifically employed machine-learning techniques to meticulously screen biomarker combinations that hold significant potential in the diagnosis of heart failure. During the subtype analysis based on the combination of the four biomarker genes, a notable discovery was made. Subtype 1 predominantly exhibited enrichment in replication and repair-related pathways. Significantly, subtype 3 was chiefly enriched in immune-related pathways, such as the TGF-β signaling pathway, the IL6-JAK-STAT3 signaling pathway, and the INF signaling pathway. This finding potentially implies that immune pathways could have a substantial impact on both the diagnosis and treatment of heart failure, thus offering novel insights into the underlying mechanisms and potential therapeutic strategies for this complex cardiovascular disorder.

Subsequently, the four possible biomarkers were examined and their relationship with heart failure was explored in conjunction with existing literature. FCN3 is part of the innate immune system and serves as a strong activator for the lectin-based complement pathway (31). Research conducted previously indicates that the levels of FCN3 expression were notably reduced in various cancerous tissues, including squamous cell lung carcinoma (32), hepatocellular carcinomas (33), and ovarian cancer (34). However, other studies have shown that lower serum levels of FCN3 could correlate with adverse outcomes in heart failure, suggesting its potential as a prognostic marker. Its expression was found to be decreased in patients with ischemic cardiomyopathy, indicating a protective role against the progression of heart failure through the regulation of immune cell infiltrations, particularly impacting neutrophils and monocytes (35, 36). SMOC2 is a protein that is secreted into the matrix and plays a role in several pathophysiological processes, including angiogenesis, tumor development, tissue fibrosis, and calcification (37–39), and can also serve as a predictive biomarker for various diseases (40–43). SMOC2 has been depicted as significantly associated with the pathophysiology of heart failure. Numerous research efforts have demonstrated that SMOC2 facilitates tissue fibrosis through the modulation of fibroblast conversion into myofibroblasts, potentially resulting in fibrosis within the lungs, kidneys, and various other organs (38, 44). Meanwhile, a reduction in SMOC2 levels has the potential to mitigate myocardial fibrosis through the suppression of the ILK/p38 signaling pathway (45). Furthermore, in studies involving rat models of heart failure, knockdown of SMOC2 resulted in improved cardiac function and attenuation of collage deposition, showcasing its potential involvement in cardiac remodeling and fibrosis. Moreover, alterations in autophagy observed with SMOC2 knockdown demonstrate its influence on cellular stress responses in heart tissue, implying that SMOC2 can be a target for therapeutic strategies in heart failure management (46, 47). Among the four diagnostic biomarkers, MNS1 is thought to play a role in chromatin dynamics and cellular maintenance, and any dysfunction in its expression or localization can impact cardiomyocyte survival, stability, and, consequently, cardiac performance and health (48). Recent studies have revealed that MNS1 could be involved in the control of meiosis and germ cell differentiation, affecting the mating and recombination processes during meiosis (49), and serving as a diagnostic indicator in heart failure research (50, 51). Similar to MNS1, there is limited research on FREM1-specific functions in heart failure, but it is hypothesized to play roles in tissue remodeling and repair processes after myocardial injury (35). Jiang et al. discovered that elevated levels of the *MNS1* gene, in conjunction with *FREM1*, might influence the development of heart failure by modulating the metabolism of bile acids, fatty acids, and heme (50). In conclusion, biomarkers such as *FCN3* and *SMOC2* exhibit potential for diagnosis, emphasizing the necessity of continued investigation into their roles as diagnostic tools and therapeutic targets in heart failure management.

Currently bioinformatic studies on HF due to the different datasets and analysis methods selected, the Hub genes obtained by each study are also different. Research has suggested three potential biomarker genes (*ASB14*, *CD163*, and *CCL5*) associated with heart failure through the traditional protein–protein interaction algorithm (52). Kong et al. reported that combined analysis using WGCNA and machine-learning algorithms (LASSO, SVM-RFE, and RF) identified *CHCHD4*, *TMEM53*, *ACPP*, *AASDH*, *P2RY1*, *CASP3*, and *AQP7* as potential biomarkers for HF based on the GSE57338 dataset (53). In addition, there have also been studies using machine-learning algorithms (LASSO regression and the SVM-RFE algorithm) based on multiple datasets to identify *SDSL* as a driver gene in patients with heart failure (54). Compared with previous methodologies, this study established an integrated analytical framework that synergizes WGCNA with machine-learning algorithms across multi-cohort datasets. Furthermore, we implemented consensus clustering analysis on rigorously screened hub genes, successfully identifying molecular subtypes characterized by distinct immune microenvironment profiles. Our results and theirs can complement each other and provide novel insights into potential clinical treatment strategies for patients with heart failure.

Significant clinical and experimental evidence indicates that HF is a complex pathophysiological condition characterized by essential roles played by myocardial remodeling, inflammatory activation, and the activation of myofibroblasts and immune cells (55). A key factor in the occurrence and progression of cardiovascular disease is immune cell infiltration (56, 57). Infiltration and activation of different myocardial immune cells cause heart inflammation, tissue damage, and ultimately heart failure (58). Single-cell RNA sequencing demonstrated that most innate immune cell subsets, including mast cells, monocytes, macrophages, neutrophils, dendritic cells, and natural killer cells, were significantly activated in mice with heart failure induced by pressure overload (59). In this research, when compared with the control group, we observed a higher quantity of DCs, mast cells, and T cells in HF samples, highlighting their significant roles in the etiology of HF. Our results confirm the results of Patella et al. and Abdolmaleki et al. who reported that mast cells are increased in HF, with a corresponding increase in T cells (60, 61). In addition, based on the four diagnostic marker genes identified in this study, we identified three unique subtypes, each exhibiting different enriched functions and pathways, alongside variations in immune cell infiltration and immunological characteristics. These findings provide a new perspective on how the immune microenvironment within heart tissues relates to the prognosis and categorization of patients with HF.

Recent studies have demonstrated that changes in the microenvironment not only cause pathological changes such as cardiomyocyte hypertrophy and energy metabolism disorders, but also indirectly stimulate tumor tissues via the influence of growth factors, cytokines, and chemokines acting through paracrine or endocrine mechanisms in the bloodstream (62, 63). Several investigations have examined the occurrence of cancer among individuals with a previous diagnosis of heart failure, revealing a confirmed heightened risk (14, 64). In a case-control study, Hasin et al. investigated the risk of cancer occurrence among patients with HF, revealing that those with HF faced a 60% increased likelihood of developing malignancies, in contrast to non-HF controls (65). The research specifically identified that lung and skin cancers were the two most prevalent new-onset malignancies in the heart failure population examined, with renal and urinary cancers following closely, as both exhibited identical hazard ratios. Essentially, all forms of cancer occurred more often except for prostate carcinoma (64). Moreover, substances released by tumors can lead to cardiovascular problems regardless of the cardiotoxicity associated with anti-cancer therapies (66, 67), but further investigation in this domain is necessary (68, 69).

The novel aspects of our study are as follows. First, through integrative bioinformatics analysis employing three distinct machine-learning algorithms, we systematically identified *FCN3*, *FREM1*, *MNS1*, and *SMOC2* as novel combinatorial biomarkers. Second, external cohort validation demonstrated that our four-gene signature exhibits robust diagnostic performance. Finally, our consensus clustering analysis revealed three distinct immune subtypes based on the expression patterns of these biomarkers, unveiling significant heterogeneity in the cardiac immune microenvironment. Our findings contribute to improving diagnostic accuracy and stratified clinical management while enhancing the clinical practicality of early heart failure detection and optimizing targeted drug treatment models. Furthermore, these immunophenotypic classifications can not only inform personalized immunomodulatory treatment strategies but also establish a foundation for precision clinical trial designs through immune-stratified patient selection.

Nonetheless, this study has certain limitations. First, since the research relied on publicly accessible datasets, the sample size was still small. More datasets are needed to validate our diagnostic model and further prospective samples for experimental assessment are necessary to ensure further validation. Second, the dataset we used comes from myocardial tissue, and insufficient validation of the peripheral blood datasets may limit the applicability of the diagnostic model. Finally, due to the lack of information on crucial clinical characteristics such as survival time, smoking, drinking, and previous therapies, it is impossible to fully assess or control for potential confounding factors in our analyses.

## Conclusion

Overall, our study revealed that *FCN3*, *FREM1*, *MNS1*, and *SMOC2* can serve as diagnostic biomarkers for HF, deepening the understanding of its pathogenesis.

## Data Availability

The original contributions presented in the study are included in the article/Supplementary Material, further inquiries can be directed to the corresponding author.
