# Supplementary figures and images for: Identification of signature genes and subtypes for heart failure diagnosis based on machine learning
Source: Front Cardiovasc Med. 2025 Apr 14;12:1492192. doi: 10.3389/fcvm.2025.1492192 (PMC12034685; doi:10.3389/fcvm.2025.1492192)

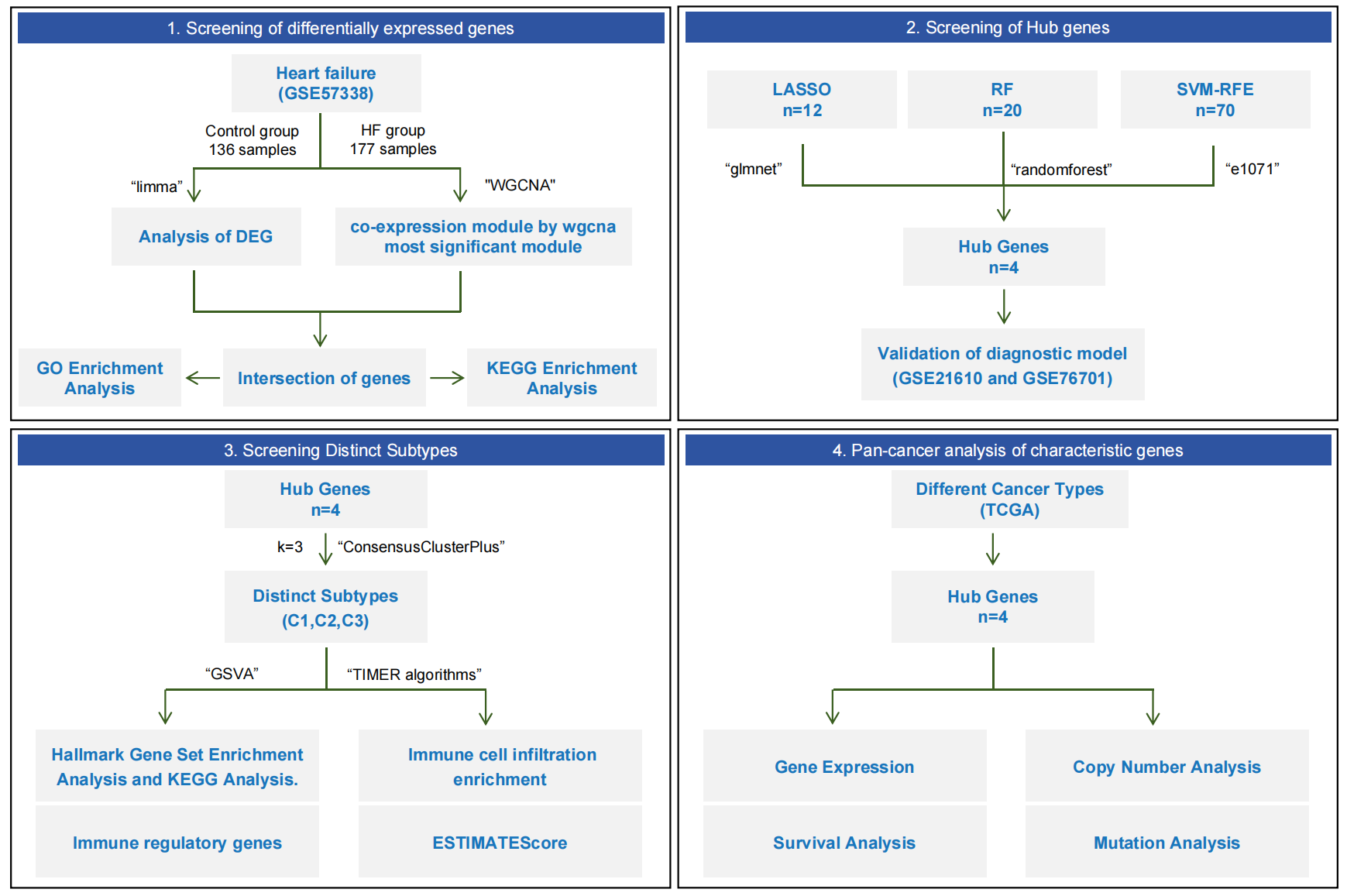

Supplement: Supplementary file 1 [file Image1.tif]
